# Investigation into Biosorption of Pharmaceuticals from Aqueous Solutions by Biocomposite Material Based on Microbial Biomass and Natural Polymer: Process Variables Optimization and Kinetic Studies

**DOI:** 10.3390/polym14163388

**Published:** 2022-08-19

**Authors:** Lăcrămioara Rusu, Cristina-Gabriela Grigoraș, Andrei-Ionuț Simion, Elena-Mirela Suceveanu, Carol Schnakovszky, Lidia Favier

**Affiliations:** 1 Faculty of Engineering, “Vasile Alecsandri” University of Bacau, 157 Calea Mărăşeşti, 600115 Bacau, Romania; 2 Ecole Nationale Supérieure de Chimie de Rennes, University of Rennes, CNRS, UMR 6226, CEDEX 7, 35708 Rennes, France

**Keywords:** biosorption optimization, Box–Behnken design, ethacridine lactate, *Saccharomyces pastorianus*, natural polymer, kinetic models

## Abstract

Biosorbtive removal of the antibacterial drug, ethacridine lactate (EL), from aqueous solutions was investigated using as biosorbent *Saccharomyces pastorianus* residual biomass immobilized in calcium alginate. The aim of this work was to optimize the biosorption process and to evaluate the biosorption capacity in the batch system. Response surface methodology, based on a Box–Behnken design, was used to optimize the EL biosorption parameters. Two response functions (removal efficiency and biosorption capacity) were maximized dependent on three factors: initial concentration of EL solution, contact time, and agitation speed. The highest values for the studied functions (89.49%, 26.04 mg/g) were obtained in the following operational conditions: EL initial concentration: 59.73 mg/L; contact time: 94.26 min; agitation speed: 297.57 rpm. A number of nonlinear kinetic models, including pseudo-first-order, pseudo-second-order, Elovich, and Avrami, were utilized to validate the biosorption kinetic behavior of EL in the optimized conditions. The kinetic data fitted the pseudo-first-order and Avrami models. The experimental results demonstrated that the optimized parameters (especially the agitation speed) significantly affect biosorption and should be considered important in such studies.

## 1. Introduction

A key turning point in scientific and technical progress worldwide was the develop-ment of pharmaceuticals, which have raised standards of living, extended life expectancy, and cured millions of people of otherwise terminal diseases [1,2]. Due to their prominence, pharmaceutical pollutants have over the past three decades increasingly been recognized as rapidly growing environmental contaminants, being discovered in almost all ecological matrices on every continent [3].

Pharmaceuticals are among the most important categories of environmental water contaminants to be concerned about, due to the fact that conventional wastewater treatment plants are not designed to completely eliminate them [4,5]. The use of many different therapeutic agents has led to a complicated problem of environmental contamination. There have been at least 11,926 pharmaceutically active compounds found in total, and 713 of these were detected in wastewater [6,7]. Meanwhile, the COVID-19 pandemic has led to a sharp increase in the generation of medical waste, according to the existing literature [2,8].

Therefore, future efforts to reduce pharmaceutical pollution are likely to prioritize and incorporate techniques and methods that have already been shown to be effective [9,10,11,12]. In order to meet current demand without negatively impacting the environment, it is crucial to provide long-term solutions based on technological, economical, and environmental conditions in a sustainable way, for example, by avoiding the direct disposal of pharmaceuticals into water sources [13,14].

Due to its simplicity of operation and design, and lack of undesirable by-products, adsorption/biosorption has been considered a promising method for aqueous matrix decontamination of pharmaceuticals [15,16].

In order to obtain low-cost sustainable biosorbents, a wide variety of materials such as microbial biomass (bacteria, cyanobacteria, fungi, yeasts, and microalgae), microbial residual biomass (resulting from fermentation processes), clays, chitosan, biochar, etc. have already been studied [2,14,17,18,19,20].

Our previous studies have also demonstrated that biosorption processes are effective for the removal of pharmaceutical compounds from aqueous solutions if a viable biosorbent is used. Biocomposite materials obtained by immobilizing or encapsulating microbial biomass in polymer matrices were successfully used as biosorbents [13,14,21,22,23].

Most of the conventional ways to examine a biosorption process include keeping constant certain variables while assessing the impact of others. This frequently used approach is not capable of effectively displaying the interplay between settings. Additionally, many experiments need to be run, which requires extensive time. Response surface methodology (RSM) is one of the suitable methods used in different fields for determination of optimal operational conditions. It is a highly helpful tool which screens the variables, serves in choosingexperimental designs, offers mathematical models that assist the understanding of the connections between the studied parameters, evaluates the models’ adequacy, and establishes the optimal conditions and appropriate values [24]. One of the RSM designs, the Box–Behnken design gives thorough details of the solution space and enables researchers to comprehend the factors influencing the output model [25,26,27,28,29,30]. It needs a reduced number of runs, can be used for obtaining maximum information with minimum time and resource requirements [25,31], and has been applied for the study of water contaminant biosorption from diverse materials including cadmium retention by metal-reducing bacterium [32], heavy metal removal with a new strain of *Pseudomonas azotoformans* [33], phytoremediation of dyes [34], etc.

The aim of this current work was to optimize several parameters impacting the biosorption of pharmaceuticals from aqueous solutions, and to assess their kinetic behavior.

Residual biomass of *Saccharomyces pastorianus*, the second-largest by-product of the brewing industry, which is considered safe, low-cost, and available throughout the year in large volumes, was chosen for biosorbent synthesis [23].

Ethacridine lactate was selected as the target molecule, as it is a widely used anti-microbial drug worldwide [22].

Response surface methodology (RSM) using the Box–Behnken design was applied, and two response functions—removal efficiency (R%) and biosorption capacity (Q mg/g)—were evaluated. Three factors affecting the biosorption process were optimized: initial concentration of EL solution, contact time and agitation speed. To the best of our knowledge, this is the first time the optimization of the system of biocomposite beads and pharmaceutical aqueous solution has been carried out, taking into account as parameters the stirring speed in addition to the initial pollutant concentration and the contact time. The physicochemical characteristics of the adsorbate (molecular mass, polarity, types of functional groups), the solution chemistry (ionic strength, pH), and the temperature can all affect the biosorption process [35]. Most of these parameters have a well-known and documented effect on the kinetics and adsorption equilibrium of various pharmaceutical contaminants. However, authors have rarely disclosed the methods used to improve the contact between the adsorbent and the water matrix. The impact of agitation on adsorption/biosorption is typically disregarded and has only been examined briefly by a small number of researchers. There is sufficient information regarding equilibrium and kinetics in the literature, but little is known about how agitation speed affects these processes [35,36,37,38,39].

The commonly used non-linear kinetic models Pseudo-first-order, pseudo-second-order, Elovich, and Avrami were utilized to validate the kinetic biosorption behavior of ethacridine lactate in the obtained optimized conditions.

## 2. Materials and Methods

### 2.1. Chemicals and Investigation Method

Analytical purity reagents required through all the conducted experiments were used as received from commercial agents, without any treatment or purification.

The 2-ethoxy-6,9-diaminoacridine monolactate monohydrate, K_2_HPO_4_, and Na_2_HPO_4_ came from Merck (Darmstadt, Germany). HCl, NaCl and ethanol were bought from the Chemical Company (Iași, Romania). NaCl and CaCl_2_ were delivered by Chempur (Piekary Ślaskie, Poland). Sodium alginate (low viscosity grade) was procured from BUCHI Laboratortechnik AG (Flawil, Switzerland).

Residual biomass of *Saccharomyces pastorianus* was donated by the brewing company Albrau (Onești, Romania) and stored at −20 °C in sealed plastic bags until use.

Distilled water was used to prepare all the solutions.

When necessary, pH corrections were made by adding NaOH (0.1 M) or HCl (0.1 M).

An EL stock solution (500 mg/L) was prepared and set aside at 4 °C until it was required for further dilutions (between 1 mg/L and 60 mg/L). These solutions were spectrophotometred at 431 nm on a UV1280 apparatus (Shimadzu, Tokyo, Japan) and served to draw a calibration curve.

All the experiments were performed in triplicate.

### 2.2. Biosorbent Preparation and Characterization

Residual microbial biomass of *Saccharomyces pastorianus* passed through a preliminary treatment consisting in thawing, multiple washes, and successive settlings and centrifugations. The latter operation was managed in a Quirumed 80-2A laboratory centrifuge (Jintan City, China) at a rotation speed of 2500 rpm twice for 10 min. A suspension (5%, *w*/*v*) was prepared from residual biomass and sodium alginate solution (1% in phosphate buffer of pH 7). Intense homogenization was then realized on a Nahita magnetic plate (Auxilab, Beriáin, Navarra, Spain). The mixture was dripped into a 2% (*w*/*v*) CaCl_2_ solution. The composite beads (hereafter SPRBA 5%) were washed with CaCl_2_ 2% and maintained in a fresh solution for 24 h at 4 °C. The biosorbent was carefully washed with distilled water prior to the biosorption experiments.

Beads’ characterization (before and after biosorption) was realized by the means of scanning electron microscopy (SEM) and Fourier-transform infrared spectroscopy (FTIR). The point of zero charge (pH_PZC_) was established.

SEM examinations were carried out with a Quanta 200 3D apparatus (FEI Europe B.V., Eindhoven, The Netherlands)). The SPRBA 5% material was initially dried for 2 h at 50 °C in a laboratory oven (Air Performance AP60 Froilabo, Paris, France) and then placed on double-adhesive carbon discs fixed on stubs. Normal secondary electron mode (SE) in a low vacuum was established for the investigation. Detection was ensured by a large field detector (LFD) at an accelerating voltage of 20 kV, a working distance of 14.5 mm, and a spot size of 5. The magnification range was between 2 mm and 20 µm.

FTIR spectra were recorded from 4000 to 400 cm^−1^ (32 scans co-added) with a resolution of 4 cm^−1^ on a Nicolet iS50 FT-IR spectrometer (Thermo Scientific, Dreiech, Germany) including a built-in ATR accessory, DTGS detector, and a KBr beam splitter. The ATR plate was cleaned with ethanol after each spectrum acquisition. Air was used for background spectrum reference, which was registered and compared with the anterior.

For the biosorbent point of zero charge determination, volumes of 25 mL of a 0.1 M NaCl solution were used as background electrolyte. The initial pH values (pH_i_) were corrected with hydrochloric acid (0.1 M) or sodium hydroxide (0.1 M) and read with a Dostmann KLH9.1 pH meter (Carl Roth, Karlsruhe, Germany). Amounts of 0.5 g of biosorbent were added to each solution. After 24 h of magnetically stirring at room temperature, the final pH values (pH_f_) were measured again. The sample pH_PZC_ point was recovered from the pH_f_ vs. pH_i_ curve.

### 2.3. Experimental Design

The Box–Behnken design (BBD) was selected for the optimization of three parameters (ethacridine lactate initial concentration, agitation speed, and biosorption time) with impact on the removal of EL from aqueous solution by biosorption on SPRBA 5% material. Their coded and actual values are given in Table 1.

The experiments were conducted with 30 mL of EL solution at pH 4 and different concentrations in contact with 1.2 g of biosorbent and agitated at different speeds on a hot plate magnetic stirrer (DLAB MS-H280-PRO, DLAB Scientific Inc., Riverside, CA, USA) for different periods of time.

EL removal efficiency and biosorption capacity were estimated with Equations (1) and (2).

(1) $$R\;\left(\%\right)=\frac{(C_{0}-C_{e})\cdot 100}{C_{0}}$$

(2) $$Q_{e}\;\left(\mathrm{mg}/g\right)=\frac{\left(C_{0}-C_{e}\right)\cdot V}{m}$$

where *C*_0_ and *C_e_* are the initial and equilibrium state concentrations (mg/L); *V* is the EL volume (L); *m* is the amount of the biosorbent (g).

The EL removal efficiency and the biosorption capacity were set as response functions for the BBD, expressed as second-order polynomial equations whose general form is represented by Equation (3).

(3) $$Y_{K}=\alpha_{0}+\alpha_{1}\cdot A+\alpha_{2}\cdot B+\alpha_{3}\cdot C+\alpha_{4}\cdot A\cdot B+\alpha_{5}\cdot A\cdot C+\alpha_{6}\cdot B\cdot C+\;\alpha_{7}\cdot A^{2}+\alpha_{8}\cdot B^{2}+\alpha_{9}\cdot C^{2}$$

where *Y_K_* is the response function; *α*_0_ is the intercept; *α*_1_–*α*_9_ are the regression coefficients; *A*, *B* and *C* are the coded independent variables.

Experimental data were processed in Design expert 13.0 software (Stat-Ease, Minneapolis, MN, USA) in terms of regression model analysis and statistical calculation.

### 2.4. Biosorption Kinetics

Kinetic experiments were carried out with 30 mL of EL solutions and 1.2 g of biosorbent in the optimized conditions established by BBD.

CAVS adsorption evaluation software, version 2.0 (Federal University of Paraná, Curitiba, Paraná, Brazil) was used to apply different nonlinear kinetic models to study the target molecules’ removal by the prepared biosorbent.

Pseudo-first-order (Equation (4)), pseudo-second-order (Equation (5)), Elovich (Equation (6)), and Avrami (Equation (7)) were applied for testing:

(4) $$Q_{t}=Q_{e}\cdot \left(1-e^{-k_{1}\cdot t}\right)$$

(5) $$Q_{t}=\frac{k_{2}\cdot Q_{e}^{2}\cdot t}{\left(1+k_{2}\cdot Q_{e}\cdot t\right)}$$

(6) $$Q_{t}=\frac{1}{\beta }\cdot \ln\left(\alpha \cdot \beta \cdot t\right)$$

(7) $$Q_{t}=Q_{e}\cdot \left(1-e^{\left(-k_{Av}\cdot t^{n_{Av}}\right)}\right)$$

The parameter significance was as follows: *Q_t_*—concentration on the solid phase at time *t*, mg/g; *Q_e_*—adsorbent capacity at equilibrium, mg/g; *k*_1_—pseudo-1st-order constant rate, 1/min; *t*—contact time, min; *k*_2_—pseudo-second-order constant rate, g/(mg·min); *β*—extent of surface coverage and activation energy for chemisorption, g/mg; *α*—initial adsorption rate, mg/(g·min); *k_Av_*—overall rate constant, 1/min; *n_Av_*—parameter related to the adsorption, without dimension.

Root mean square error (RMSE), Marquardt’s percent standard deviation (MPSD), hybrid fractional error function (HYBRID), chi-square (χ^2^), and coefficient of determination (R^2^) calculated by CAVS software served to assess the adequacy of the kinetic model.

## 3. Results and Discussion

### 3.1. Biosorbent Preparation and Characterization

The first aim of this experimental work consisted in the preparation of a new material possessing the properties of an adsorbent. Since the raw material was in the form of a residual microbial biomass (*Saccharomyces pastorianus*), it was necessary to insure its stability. Therefore, an immobilization step was conducted with sodium alginate chosen to this purpose since it is known as a non-toxic, biodegradable, and highly available natural polymeric matrix. Composed of (1-4)-linked β-d-mannuronic acid and α-l-guluronic acid, sodium alginate is able to form a network structure with divalent cations including calcium [40], and it successfully provided good immobilization of the *Saccharomyces pastorianus* residual biomass.

SPRBA 5% synthesized beads had a regular, spherical form and a dark white nuance. Their mean diameter was of 3.159 ± 0.018 mm.

In a second step, the obtained biosorbent was characterized by different techniques.

**Figure 1:**
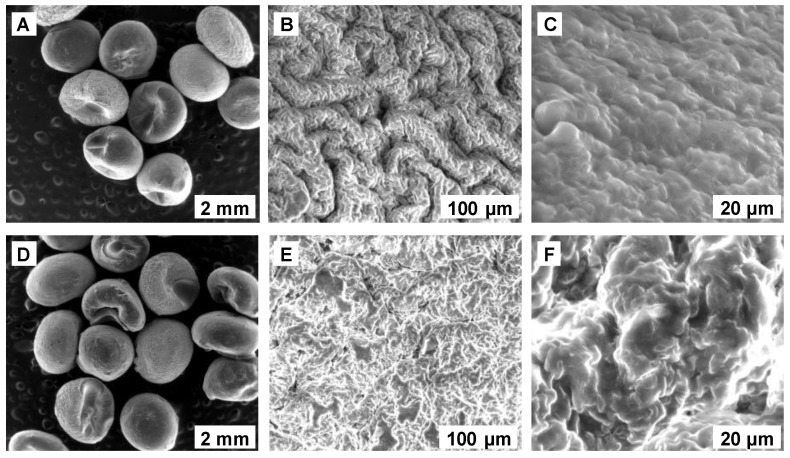
SEM pictures of the prepared composite material, SPRBA 5%, (**A**–**C**) before and(**D**–**F**) after EL biosorption (SPRBA 5%-*Saccharomyces pastorianus* residual biomass immobilized in calcium alginate with 5% dry mass).

SEM analysis, before and after EL biosorption, is depicted in Figure 1.

A smooth external surface was observed, presenting some abnormalities, assignable to the preparation process. The internal morphology displayed a uniform, porous structure with rolling predispositions before EL biosorption, and with agglomerative tendencies after finalizing the process. The encountered modifications confirmed the retention of the tested pollutant.

**Figure 2:**
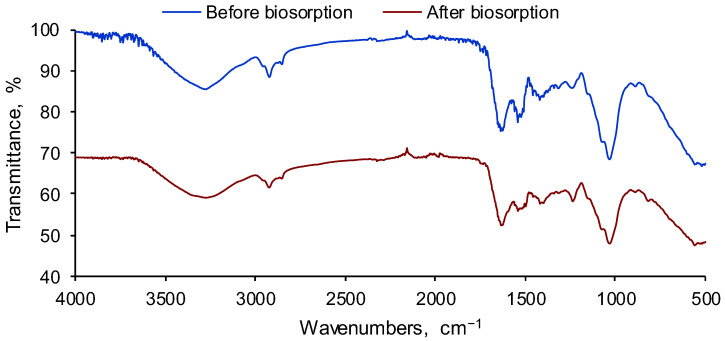
FTIR spectra of SPRBA 5% material before and after EL biosorption.

Functional groups existing in the SPRBA 5% biosorbent were inspected by FTIR investigation. Spectra visible in Figure 2 disclose the presence of the inert alginate matrix.

At frequencies between 3000 cm^−1^ and 3200 cm^−1^, hydroxyl vibrations were detected. –CH aliphatic stretching vibration was noted at 2920 cm^−1^ [41]. Bands from 1600 cm^−1^ to 1400 cm^−1^, particular the asymmetric and symmetric stretching vibrations of carboxyl ions [42] of C–O (1100 cm^−1^ to 900 cm^−1^) and mannuronate and guluronate residues (1030 cm^−1^) [43], defined the natural polymer used for the immobilization of *Saccharomyces pastorianus* residual biomass. A –CH_2_ bending vibration close to 1000 cm^−1^ was also detectable. Resemblances in the FTIR spectra interpretations corresponded with those disclosed by other researchers [44]. Peaks of 1630 cm^−1^ and 1540 cm^−1^ can be ascribed to amide I and amide II. From 1300 cm^−1^ and 1200 cm^−1^ could be observed bands for amide III (proteins) and PO_2_^−^ (phosphorylated proteins and phospholipids), probably result of the yeast integrated into the polymeric material [45]. These findings validate the idea that residual biomass of *Saccharomyces pastorianus* was well-incorporated in the resulted adsorbent material.

Spectra collected after biosorption show that EL signals were overlapped by the biosorbent functional groups. This was the case for peaks from 3500 cm^−1^ to 3100 cm^−1^, particular to the N–H asymmetric and symmetric stretching vibrations of aromatic amine and hydrogen-bonded N–H bands. Moreover, the peak from approximatively 1630 cm^−1^ was found to be typical for C=N vibrations of the EL acridine ring [46]. Therefore, it can be concluded that the SPRBA 5% biosorbent retained the studied pollutant from the aqueous solutions.

**Figure 3:**
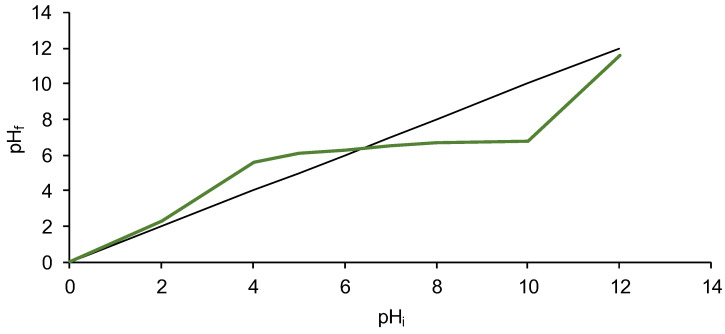
pH_PZC_ of SPRBA 5% biosorbent (pH_f_, final pH; pH_i_, initial pH).

The final experiment conducted for SPRBA 5% biosorbent characterization was represented by the establishment of the point of zero charge. As we reported in previously published papers [13,14,21,22,23] and as described in the literature [47], this point is the pH value at which the charge of the positive surface sites is equal to that of the negative surface sites, the biosorbent surface charge being null. pH_PZC_ is an important indicator for whether the surface charge is negative (pH lower than pH_PZC_) or positive (pH higher than pH_PZC_). Figure 3 illustrates two augmentations of pH_f_, from 2.30 to 5.60 when the initial pH increased from to two to four, and from 6.80 to 11.60 for an increase of the initial pH from 10 to 12. When pH_i_ was between four and ten, only very small variations of the final pH value were recorded (6.10 to 6.80) which denotes that in this period neither the environmental addition of acid nor of base affected the final pH. As depicted in Figure 3, the pH_PZC_ of the synthesized biosorbent was 6.5.

### 3.2. Box–Behnken Design and Model Validation

#### 3.2.1. Box–Behnken Design

Another aim of this work was to study the usability of the synthesized biosorbent for removing drugs from aqueous solutions, with ethacridine lactate selected as the model molecule. The influence of various factors is known to affect the biosorption process of this compound on SPRBA 5% prepared material, which was the object of our previous papers [13,22,23]. According to these already reported outcomes, the most convenient results were attained when the biosorbent dosage was established at 2 g/L, the pH of EL solutions set at four, and the operating temperature was ambient.

The Box–Behnken design is a rotatable second-order design useful for the optimization of experiment numbers required for the determination of interactions occurring between studied parameters and their impact on the process. Three levels of variation were considered for three other factors recognized as important in the development of a biosorption setup: (A) EL initial concentration, (B) agitation speed, and (C) time. Data acquired for removal efficiency and biosorption capacity when using these input variables are tabulated in Table 2.

Investigation of the response function values shows that the predicted values were very similar to those obtained experimentally, indicating that the quadratic mathematical models expressed by Equation (8) for removal efficiency and by Equation (9) for biosorption capacity were able to describe the relation between the input factors and the response functions.

(8) $$Y_{R}=81.5433+4.0240\cdot A+3.4675\cdot B+28.5095\cdot C+1.4821\cdot A\cdot B+2.5727\cdot A\cdot C\;-3.1879\cdot B\cdot C-7.9556\cdot A^{2}-1.7699\cdot B^{2}-22.1559\cdot C^{2}$$

(9) $$Y_{Q_{e}}=16.0704+6.8854\cdot A+0.7575\cdot B+5.7668\cdot C+0.6109\cdot A\cdot B+3.3709\cdot A\cdot C\;-0.6516\cdot B\cdot C-1.1569\cdot A^{2}-03035\cdot B^{2}-4.3648\cdot C^{2}$$

Positive signs within the above equations suggest a reciprocal effect of the factors, while negative signs imply an antagonistic impact [48].

**Figure 4:**
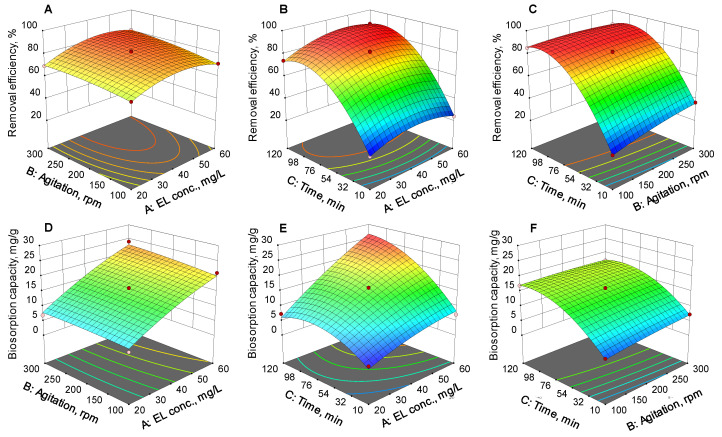
Three-dimensional response surface models of (**A**–**C**) removal efficiency and (**D**–**F**) biosorption capacity response functions.

Figure 4 illustrates the three-dimensional response surfaces for the two response functions. For each investigation, two of the studied parameters were used simultaneously while maintaining the third constant at zero.

The observable nonlinear nature of the results shows that there were interactions between the input variables and the output functions. It is evident that both the removal efficiency and the biosorption capacity were influenced by an increase of the EL concentration, of the agitation speed, and of the contact time. This effect can be explained by the fact that, under agitation conditions, the biosorbent active sites are more easily accessible for the EL molecule. Likewise, greater contact time between SPRBA 5% beads and the pollutant encountered in the aqueous solutions ensured better biosorption. It can be observed that agitation had an essential role in shortening the biosorption time. In static conditions [23] after a contact time of 5 h, the removal efficiency and biosorption capacity of unagitated solutions were lower than those obtained under agitation (81.26% vs. 90.72% and 24.30 mg/g vs. 26.98 mg/g) for the biosorption of the same pollutant existing in an aqueous solution with the same pH and concentration,.

Similar findings have been highlighted by other researchers. For example, Sadrnia et al. [49] optimized the removal of doxorubicin by retention on adsorbent nanoribbons and showed that time (along with pH, adsorbent weight, and temperature) was a key factor in the process. Babas et al. [50] dedicated a recent study to the adsorption of sofosbuvir on activated carbon derived from argan shell residue, and indicated that pollutant removal was highly affected by parameters such as pH, adsorbent amount, and target molecule concentration. Ajebli et al. [51] revealed in their work that tenofovir elimination by adsorption on activated carbon obtained from maize cobs was optimized by Box–Behnken design, and that parameters such as pH, adsorbent mass, and initial drug concentration had a decisive impact on the process.

Analysis of variance (ANOVA) (Table 3 and Table 4) was employed to establish the statiscal significance of EL concentration, agitation speed, and time.

The modeled high F-values of 2279.93 (for removal efficiency response function) and 68.96 (for biosorption capacity response function) revealed that both models were significant, and that there was only 0.01% chance that *F*-values this large could occur due to noise.

Overall, *p*-values inferior to 0.05 suggest that model terms are significant. For removal efficiency, it appeared that all three tested parameters (EL initial concentration, agitation speed, and time) as well as their interactions and their quadratic forms were of great importance for the process. In the case of biosorption capacity, greater impact was observed due to EL initial concentration, time, their interaction, and the quadratic form of the last cited parameter.

Summary statistics indicated a good fit and high significance for the obtained models. Correlation coefficients (R^2^) of 0.9997 for the removal efficiency model and 0.9888 for the biosorption capacity model were calculated. Adjusted correlation coefficients (Adj. R^2^) and predicted correlation coefficients (Pred. R^2^) had similar values (for the removal efficiency model: Adj. R^2^ = 0.9992 and Pred. R^2^ = 0.9957; for the biosorption capacity model: Adj. R^2^ = 0.9745 and Pred. R^2^ = 0.8220).

#### 3.2.2. Models Validation

One of the major advantages of using a response surface methodology such as Box–Behnken design resides in the possibility of obtaining optimized values of the tested parameters and predicting what results should be expected.

In our specific situation, based on the experimental data utilized, Design Expert software was able to generate multiple possibilities with high desirability. Among these included the following recommended values for the implied factors: A = 59.7341 mg/L; B = 297.566 rpm; C = 94.2582 min. Under these conditions, the removal efficiency should be 89.4855% and the biosorption capacity should be 26.04 mg/g.

In order to confirm this suggested resolution, three different experiments were conducted with an EL solution at a concentration of 60 mg/L under an agitation speed of 300 rpm for 94 min. A removal efficiency of 90.7195% ± 0.8341% and a biosorption capacity of 26.9828 mg/g ± 0.6802 mg/g were calculated. Good agreement between predicted and verified values was clearly apparent, sustaining the observation that the mathematical models well fitted the investigational data and were highly compatible and reliable.

### 3.3. Biosorption Kinetics

The experiments were carried out at pH 4, with 30 mL of EL and 2 g/L SPRBA 5%. The initial EL concentration and the agitation speed were those suggested by the Box–Behnken design, respectively 60 mg/L and 300 rpm.

For the examination of the process control mechanism, several different equations were applied for modelling the kinetics of EL biosorption on the prepared material. Pseudo-first-order, pseudo-second-order, Elovich, and Avrami nonlinear forms were tested, in order to establish which was appropriate to describe the EL biosorption.

Pseudo-first-order modelling offers the possibility of discovering the values of the time-scaling factor, which is able to estimate the time taken to reach the equilibrium state and the amount of adsorbed pollutant at equilibrium [52]. Pseudo-second-order kinetics is another widely used model. It relies on the supposition that adsorption implicates chemical interaction, with electrostatic interactions, etc., happening between the molecule to be adsorbed and the biosorbent surface. Initial concentration of pollutant, enthalpy and entropy fluctuations must also be taken into consideration as influencing the biosorption process [53]. The Elovich kinetics model assumes that a chemical mechanism leads the adsorption process, and seems to be applicable for systems evolving heterogeneous adsorbing surfaces [54,55]. The Avrami model postulates that the surface of the active sites is the place of reaction between the adsorbate and adsorbent. Its foremost factors are the two constants included in Equation (7), the latter of these representing shifts of the adsorption mechanisms dependent on time and operating temperature [56,57,58].

**Figure 5:**
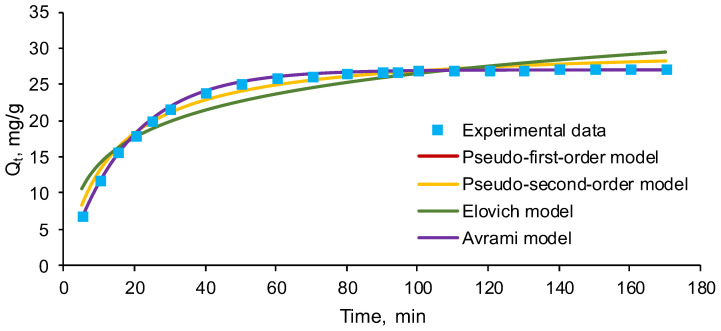
Kinetic models for the biosorption of EL on SPRBA 5% synthesized biosorbent (*Q_t_*—concentration on the solid phase at time *t*).

Figure 5 depicts the above-mentioned kinetics models fitted to the experimental data that was recorded.

The biosorption kinetic parameters recovered from the graphical plots, and their statistical error functions, are specified in Table 5.

From Figure 5 and Table 5, it can be seen that the biosorption capacities reported by pseudo-first-order and Avrami kinetics, followed closely by that of pseudo-second-order model, were comparable to those obtained experimentally and to those suggested by the Box–Behnken design. Furthermore, the low values of statistical error functions confirm likewise the idea that these models are appropriate for explaining the EL biosorption on SPRBA 5%. Certain previous research [59,60] inspected the pseudo-first-order and pseudo-second order kinetics and revealed that the adsorption is described by the pseudo-first-order equation at elevated initial pollutant concentrations, while the pseudo-second-order equation is more suitable to define the adsorption behavior at reduced concentrations. This supposition corroborates with conclusions drawn by researchers who showed that, for example, the adsorption of triclosan on simple-walled carbon nanotubes [61] can be interpreted by the pseudo-first-order kinetic model. Other papers have suggested that the experimental data recorded for adsorption of various drugs on different adsorbent materials are matched more easily by the pseudo-second-order kinetic model (e.g., adsorption of doxorubicin hydrochloride on mango seeds used for carbon activation [62], removal of tetracycline using a new magnetic nanohybrid adsorbent [63], retention of ibuprofen and naproxen on different composites [64], elimination of carbamazepine by adsorption on chitosan magnetic nanocomposite [65]).

## 4. Conclusions

Removal of ethacridine lactate from aqueous matrices was investigated using *Saccharomyces pastorianus* residual biomass immobilized in calcium alginate as biosorbent. SEM and FTIR analyses were used to determine the morphology and surface functionalities of the synthesized biocomposite material before and after biosorption. The point of zero charge of the prepared biosorbent was also established.

Box–Behnken design was applied to optimize three process variables (EL initial concentration, agitation speed, and contact time) in order to maximize two response functions (removal efficiency and biosorption capacity). The validated results demonstrate that the optimized parameters significantly affected the biosorption, and should be considered important in such studies.

The biosorption capacities reported for pseudo-first order and Avrami kinetics, followed closely by the results of the pseudo-second-order model, were comparable to the results obtained experimentally and those suggested by the Box–Behnken design.

Biocomposite materials based on microbial residual biomass and natural polymers can be effective biosorbents for pharmaceutical removal from aqueous matrices.

In perspective, the benefits of optimizing process variables can be efficiently exploited for application in different biosorption processes.

## Figures and Tables

**Table 1:** Actual and coded levels for Ethacridine lactate biosorption on SPRBA 5%.

Factors Name	Factors Code	Factors Level		
		−1	0	+1
Ethacridine lactate initial concentration, mg/L	A	20	40	60
Agitation speed, rpm	B	100	200	300
Biosorption time, min	C	10	65	120

**Table 2:** Independent variables and experimental and predicted removal efficiency and biosorption capacity of ethacridine lactate biosorption on SPRBA 5%.

Run	Independent Variables						EL Removal Efficiency, %		Biosorption Capacity, mg/g	
	A		B		C					
	Coded Value	Actual Value	Coded Value	Actual Value	Coded Value	Actual Value	Experimental Value	Predicted Value	Experimental Value	Predicted Value
1	−1	20	−1	100	0	65	65.91	65.81	6.49	7.58
2	0	40	0	200	0	65	81.61	81.54	16.04	16.07
3	0	40	0	200	0	65	81.78	81.54	16.11	16.07
4	−1	20	0	200	−1	10	21.28	21.47	2.10	1.27
5	+1	60	0	200	−1	10	23.76	24.37	7.06	8.30
6	+1	60	−1	100	0	65	71.41	70.89	21.11	20.13
7	0	40	0	200	0	65	81.11	81.54	16.11	16.07
8	0	40	−1	100	−1	10	22.55	22.45	4.48	4.23
9	0	40	+1	300	−1	10	36.48	35.76	7.19	7.04
10	−1	20	0	200	+1	120	73.96	73.34	7.29	6.06
11	0	40	0	200	0	65	81.12	81.54	15.94	16.07
12	+1	60	+1	300	0	65	80.69	80.79	23.95	22.86
13	0	40	+1	300	+1	120	86.31	86.41	17.02	17.27
14	0	40	−1	100	+1	120	85.13	85.85	16.91	17.06
15	+1	60	0	200	+1	120	86.73	86.54	25.74	26.57
16	0	40	0	200	0	65	82.10	81.54	16.14	16.07
17	−1	20	+1	300	0	65	69.26	69.78	6.89	7.87

**Table 3:** ANOVA of quadratic model for EL removal efficiency.

Source	Sum of Squares	DF	Mean Square	*F*-Value	*p*-Value	Observation
Model	9272.38	9	1030.26	2279.93	<0.0001	Highly significant
A	129.54	1	129.54	286.67	<0.0001	Highly significant
B	96.19	1	96.19	212.87	<0.0001	Highly significant
C	6502.34	1	6502.34	14389.38	<0.0001	Highly significant
AB	8.79	1	8.79	19.44	0.0031	Insignificant
AC	26.48	1	26.48	58.59	0.0001	Significant
BC	40.65	1	40.65	89.96	<0.0001	Highly significant
A^2^	266.50	1	266.50	589.74	<0.0001	Highly significant
B^2^	13.19	1	13.19	29.19	0.0010	Significant
C^2^	2066.88	1	2066.88	4573.91	<0.0001	Highly significant
Residual	3.16	7	0.4519			
Lack of Fit	2.42	3	0.8081	4.37	0.0940	Insignificant
Pure Error	0.7390	4	0.1847			
Cor Total	9275.55	16				

**Table 4:** ANOVA of quadratic model for biosorption capacity.

Source	Sum of Squares	DF	Mean Square	*F*-Value	*p*-Value	Observation
Model	788.47	9	87.61	68.96	<0.0001	Highly significant
A	379.27	1	379.27	298.55	<0.0001	Highly significant
B	4.59	1	4.59	3.61	0.0990	Insignificant
C	266.05	1	266.05	209.42	<0.0001	Highly significant
AB	1.49	1	1.49	1.18	0.3142	Insignificant
AC	45.45	1	45.45	35.78	0.0006	Significant
BC	1.70	1	1.70	1.34	0.2855	Insignificant
A^2^	5.64	1	5.64	4.44	0.0732	Insignificant
B^2^	0.3879	1	0.3879	0.3053	0.5978	Insignificant
C^2^	80.22	1	80.22	63.14	<0.0001	Highly significant
Residual	8.89	7	1.27			
Lack of Fit	8.89	3	2.96	474.96	<0.0001	Highly significant
Pure Error	0.0249	4	0.0062			
Cor Total	797.36	16				

**Table 5:** Kinetic parameters of EL biosorption on SPRBA 5% beads.

Kinetic Model		Pseudo-First-Order	Pseudo-Second-Order	Elovich	Avrami
Kinetic Parameters	*Q_e_*	26.9978	30.3847		26.9978
	*k*_1_	0.0559			
	*k*_2_		0.0002		
	*α*			6.3717	
	*β*			0.1784	
	*k_Av_*				0.8680
	*n_Av_*				0.0645
Statistical Error Function	RMSE	0.1868	0.7605	1.7131	0.1868
	MPSD	1.5637	5.6877	14.2008	1.6066
	HYBRID	0.2695	3.8475	21.4119	0.2845
	Χ^2^	0.0525	0.6845	3.3903	0.0525
	R^2^	0.9988	0.9817	0.9071	0.9988

## Data Availability

Not applicable.
